# Risk Factors for Mortality in Sepsis Patients without Lactate Levels Increasing Early

**DOI:** 10.1155/2023/6620157

**Published:** 2023-02-24

**Authors:** Miao He, Jialin Huang, Xingxing Li, Shunda Liang, Qing Wang, Hong Zhang

**Affiliations:** ^1^Department of Emergency Medicine, The First Affiliated Hospital of Anhui Medical University, Hefei 230022, China; ^2^Department of General Practice, The First Affiliated Hospital of Anhui Medical University, Hefei 230022, China; ^3^Graduate School of Anhui University of Traditional Chinese Medicine, Hefei 230022, China

## Abstract

**Objectives:**

Our purpose was to investigate the influencing factors for mortality in sepsis patients without lactate levels increasing in the early stage.

**Methods:**

We conducted a retrospective observational study involving 830 adult sepsis patients admitted to ICU. We calculated time-weighted lactate (LacTW), a dynamic value that incorporates both the magnitude of change and the time interval of such change, to represent lactate levels in the first 24 hours. ROC curve was used to find the cutoff of LacTW for predicting mortality, and the influencing factors for lactate levels and mortality in the low lactate group were further studied. The primary outcome was hospital mortality.

**Results:**

Among 830 patients, LacTW > 1.975 mmo/L was found to be the cutoff threshold for predicting mortality (AUC = 0.646, *P* < 0.001). The following indexes related to organ dysfunction influenced LacTW: acute physiology and chronic health evaluation II (APACHE II) score (*P* < 0.001), activated partial thromboplastin time (APTT) (*P* = 0.002), total bilirubin (*P* = 0.012), creatinine (*P* = 0.037), with hypotension (*P* < 0.001), chronic kidney disease (*P* = 0.013), and required continuous renal replacement therapy (CRRT) (*P* < 0.001). Of the 394 patients in the low lactate group, age (*P* = 0.002), malignancy (*P* < 0.001), lactate dehydrogenase (*P* = 0.006), required treatment such as mechanical ventilation (*P* < 0.001), CRRT (*P* < 0.001), vasoactive drugs (*P* < 0.001), and glucocorticoid (*P* < 0.001), and failure to reach the target fluid resuscitation of 30 ml/kg within 6 hours (*P* = 0.003) were independently associated with hospital mortality.

**Conclusions:**

Due to the lower incidence of early organ dysfunction, lactate levels are not increased or delayed in some septic shock patients in the early stage, thus affecting the alertness of clinicians and the timeliness and adequacy of fluid resuscitation, and finally affects the prognosis.

## 1. Introduction

Sepsis and septic shock are essential global emergency and critical care medicine issues. More than one million people suffer from sepsis worldwide each year, causing at least 1/6 to 1/3 of deaths. Improving prevention, identification, and treatment of sepsis is a global health priority [1–4].

Hyperlactatemia is closely related to disease severity and prognosis in patients with severe sepsis or septic shock [5–7]. Sepsis patients are complicated with hyperlactatemia due to tissue hypoperfusion, excess lactate production, and decreased lactate clearance caused by hepatic and renal insufficiency. However, it does not entirely represent tissue hypoxia. The concept that lactate is merely a metabolic waste product has now evolved to the point that lactate is seen as an energy shuttle [8]. And hyperlactatemia may indicate adaptive responses to the metabolic processes of severe infections and treatment [9–11]. A decrease in lactate level with fluid resuscitation in septic shock may indicate clinical improvement, so lactate clearance can help to evaluate the overall response. However, it responds too slowly to guide acute changes in treatment. Therefore, hyperlactatemia should not be seen as a problem but as a warning of altered cellular function, and therapy should not be guided by a single lactate indicator [12, 13].

A secondary analysis compared the prognosis of patients who met the new definition of septic shock in sepsis3.0 [14] with those who did not meet the new diagnostic criteria but met the previous one. In this study, low lactate level was the main reason patients did not meet the new diagnostic criteria. Although patients with high lactate levels meet the new diagnostic criteria and have a significantly higher mortality rate, patients with low lactate levels also have a mortality rate of 14.4%, which is considerably higher than the incidence of serious diseases such as stroke and heart disease [15]. At present, multiple studies focus on patients with hyperlactatemia because elevated lactate level was well established as an independent predictor of mortality in patients with sepsis. But there exist clinical cases of patients diagnosed with sepsis or septic shock without lactate levels increasing in the early stage despite clinical deterioration. This study aimed to investigate the risk factors of mortality in patients with sepsis or septic shock without early lactate level elevation.

## 2. Materials and Methods

### 2.1. Study Design

Our study retrospectively collected data from patients diagnosed with sepsis or septic shock and admitted to the EICU or ICU in the First Affiliated Hospital of Anhui Medical University from May 2019 to March 2022, which had been approved by the Clinical Medical Research Ethics Committee of our hospital (Registration Number: PJ-2022-11-18). A total of 830 patients were enrolled in the study.

### 2.2. Inclusion and Exclusion Criteria

#### 2.2.1. Inclusion Criteria

(1) Be diagnosed with sepsis or septic shock: in accordance with the International Guidelines for the Management of Sepsis and Septic Shock (sepsis 3.0) [14, 16]; (2) adult patients (age ≥18 years); (3) stay in ICU for at least 24 hours.

#### 2.2.2. Exclusion Criteria

(1) Did not meet the diagnosis criteria of sepsis and septic shock; (2) under the age of 18; (3) the length of ICU stay did not exceed 24 hours; (4) case data were incomplete.

The primary outcome was hospital mortality.

### 2.3. Data Collection

Baseline data including age, gender, weight, smoking history, drinking history, and underlying diseases such as hypertension, diabetes, chronic liver disease, chronic kidney disease, chronic respiratory disease, cardiovascular disease, autoimmune disease, malignancy, and cerebrovascular disease were collected. We also collected blood pressure on admission to the ICU, fluid resuscitation volume, and albumin infusion volume at 3 hours, 6 hours, 12 hours, and 24 hours, and treatment measures during ICU stay such as the use of vasoactive drugs, use of glucocorticoid, mechanical ventilation, continuous renal replacement therapy (CRRT), etc.

Clinical laboratory data included: blood routine: white blood cell count (WBC), neutrophil ratio, hemoglobin (Hb), platelet count (PLT), red blood cell distribution width (RDW), and hematocrit (HCT); anticoagulant function: prothrombin time (PT), activated partial thromboplastin time (APTT), international normalized ratio (INR), and prothrombin time activity (PTA); liver function: total bilirubin (TBIL), alanine aminotransferase (ALT), aspartate aminotransferase (AST), and lactate dehydrogenase (LDH); renal function: urea nitrogen (BUN), creatinine (CRE), and estimated glomerular filtration rate (eGFR); myocardial indexes: creatine kinase (CK), creatine kinase isoenzyme (CK-MB), troponin, and myoglobin; electrolytes: serum potassium, serum sodium, serum calcium, serum phosphorus, and serum bicarbonate concentration (HCO3); inflammatory indicators: procalcitonin (PCT) and C-reactive protein (CRP); all lactates within 24 hours after ICU admission. Acute physiology and chronic health evaluation II (APACHE II) score, sepsis-related organ failure assessment (SOFA) score, and Glasgow Coma Scale (GCS) score when entering ICU were used to evaluate the severity of disease and organ dysfunction. In addition, the length of hospital stay and ICU stay were collected, and the clinical outcome was hospital mortality.

### 2.4. Statistical Analysis

We applied the time-weighted lactate value (LacTW) within 24 hours after ICU admission to represent lactate level in the early stage. The LacTW is calculated as follows: the average value of lactate at different time points multiplied by the time interval, the sum of these lactate weighted values, and then divided by the total time. This approach is based on the model established by Finney and colleagues in blood glucose control [17, 18]. Unlike in randomized, controlled trials, it is impossible to specify the absolute time interval for lactate measurement in the retrospective clinical study. As a comprehensive parameter that reflects the degree and duration of lactic acidosis, LacTW effectively avoids the potential impact of monitoring deviation caused by more frequent blood lactate monitoring in more severe patients. So, it may better represent the actual lactic acid level than the arithmetic means during hospitalization and a single lactate value at admission.

SPSS 26.0 statistical software and GraphPad Prism 9 was used to analyze the data. The Shapiro‒Wilk (SW) test was used to test the normality of the distribution. Data conforming to the normal distribution were expressed as mean ± standard deviation (*x* ± *s*), and a *t*-test was used to compare groups. Non-normally distributed data were expressed as median (interquartile range), and a nonparametric test was used for comparison between groups. Frequencies and percentages were used to describe categorical variables such as gender, underlying disease, mechanical ventilation, use of vasoactive drugs, etc., and were analyzed by chi-square tests. Simple correlation analysis (*T*-test, nonparametric test, chi-square test, etc.) was performed on the potential risk factors for hospital mortality in patients with sepsis or septic shock, and the statistically significant variables were analyzed by binary logistic regression analysis to obtain the risk factors for predicting mortality. The ROC curve was used to calculate the optimal time-weighted lactate value for predicting mortality. We divided all the participants into a high lactate group and a low lactate group according to the cutoff of LacTW, and further analyzed the differences between the two groups. And the risk factors for lactate levels and mortality in the low lactate group were analyzed by subgroup analysis. The *P* value less than 0.05 was considered statistically significant.

## 3. Results

830 patients with sepsis or septic shock were enrolled in this study, with an average age of 63 years, including 521 males and 309 females. 418 patients died during hospitalization, with an overall mortality rate of 50.36%.

We compared the baseline data and clinical characteristics between the nonsurvivors and the survivors among all patients. There were significant differences in age, length of stay in ICU, complicated with malignancy, two or more underlying diseases, scores representing disease severity, and requirement of special treatments such as mechanical ventilation, vasoactive drugs, glucocorticoid, and CRRT (Table 1). As for clinical indicators, whether the fluid resuscitation volume at 6H reached the target volume was statistically significant. There were significant differences in Hb, RDW, HCT, PT, INR, PTA, BUN, LDH, CK-MB, eGFR, serum potassium, serum phosphorus, troponin, myoglobin, PCT, initial blood lactate value, and LacTW between the nonsurvivors and the survivors (Supplementary Table 1).

In the multivariate logistic regression analysis (Table 2), the following indicators were statistically significant in predicting mortality for patients with sepsis and septic shock: age (OR = 1.022, 95% CI: 1.008–1.036, *P* = 0.002), length of hospital stay (OR = 0.962, 95% CI: 0.950–0.973, *P* < 0.001), APACHE II score (OR = 1.048, 95% CI: 1.007–1.092, *P* = 0.022), PCT (OR = 0.990, 95% CI: 0.983–0.997, *P* = 0.007), serum phosphorus (OR = 1.292, 95% CI: 1.04–1.605, *P* = 0.021), LacTW (OR = 1.171, 95% CI: 1.012–1.355, *P* = 0.034), complicated with malignancy (OR = 2.799, 95% CI: 1.677–4.673, *P* < 0.001), use of vasoactive drugs (OR = 0.261, 95% CI: 0.134–0.507, *P* < 0.001), use of glucocorticoid (OR = 0.391, 95% CI: 0.259–0.592, *P* < 0.001), mechanical ventilation (OR = 0.299, 95% CI: 0.17–0.526, *P* < 0.001), CRRT (OR = 0.34, 95% CI: 0.212–0.546, *P* < 0.001), and fluid resuscitation volume within 6 hours more than 30 mL/kg after admission to ICU (OR = 0.192, 95% CI: 0.080–0.465, *P* < 0.001).

**Figure 1:**
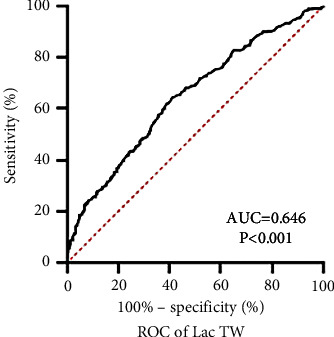
ROC curve of LacTW for predicting mortality. Note. The cutoff value of LacTW for predicting mortality was 1.975 mmol/L, and it has a sensitivity of 64.1% and a specificity of 59.2% (AUC = 0.646, *P* < 0.001, and 95% CI: 0.609–0.683).

As for LacTW, the ROC curve showed that the highest Youden value corresponded to a LacTW value of 1.975 mmo/L, with a sensitivity of 64.1% and a specificity of 59.2% (AUC = 0.646, *P* < 0.001, 95% CI: 0.609–0.683) for predicting mortality in patients with sepsis or septic shock (Figure 1).

In order to further study the indicators affecting lactate level, we conducted multiple linear regression analysis (Table 3), the following indexes had statistically different effects on LacTW: APACHE II score (*b* = 0.066, *t* = 3.607, *P* < 0.001), APTT (*b* = 0.016, *t* = 3.058, *P* = 0.002), TBIL (*b* = 0.005, *t* = 2.517, *P* = 0.012), CRE (*b* = −0.002, *t* = −2.088, *P* = 0.037), with refractory hypotension (*b* = −0.819, *t* = −3.837, *P* < 0.001), combined with chronic kidney disease (*b* = −0.923, *t* = −2.484, *P* = 0.013), and required CRRT (*b* = −1.356, *t* = −5.660, *P* < 0.001). Unexpectedly but reasonably, almost all of these are indicators related to organ dysfunction.

Based on LacTW ≤1.975 mmol/L, patients were divided into the high lactate group (*n* = 436) and the low lactate group (*n* = 394). There were no significant differences in baseline data, including gender, age, weight, history of smoking and drinking, and underlying diseases between the two groups (Table 4). The fluid resuscitation volume in the high lactate group was significantly higher than that in the low lactate group at four time points (3H, 6H, 12H, and 24H) within 24 hours after admission to ICU. With the recommended target fluid resuscitation volume of 30 ml/kg, there were statistically significant differences in whether the fluid resuscitation volume reached the target volume of the first 6H, 12H, and 24H between the two groups (Table 4). Among them, the mortality of the high lactate group was significantly higher than that of the low lactate group. However, the mortality rate in the low lactate group was still as high as 38.1%. Indicators related to organ dysfunction affecting lactate levels listed in Table 3, were significantly different between the two groups (Table 5). Therefore, it can be speculated that the low incidence of early organ dysfunction leads to the absence or delayed increase of lactate levels in these patients.

**Figure 2:**
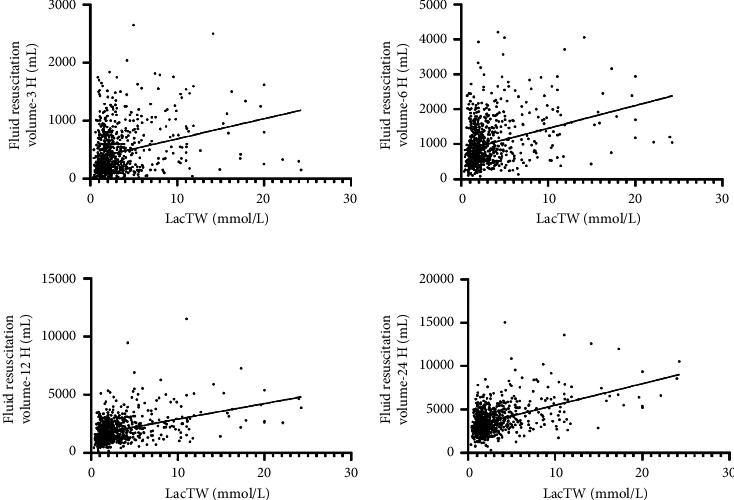
The linear correlation between LacTW and fluid resuscitation volume. Note. LacTW: time-weighted lactate. (a) The linear correlation between LacTW and fluid resuscitation volume at 3 hours after ICU admission: positive correlation, *R*^2^ = 0.076, (b) the linear correlation between LacTW and fluid resuscitation volume at 6 hours after ICU admission: positive correlation, *R*^2^ = 0.102, (c) the linear correlation between LacTW and fluid resuscitation volume at 12 hours after ICU admission: positive correlation, *R*^2^ = 0.157, and (d) the linear correlation between LacTW and fluid resuscitation volume at 24 hours after ICU admission: positive correlation, *R*^2^ = 0.225.

We conducted a subgroup analysis on the factors influencing the mortality of patients in the low lactate group. Supplementary Table 2 shows the clinical characteristics of hospital survivors and nonsurvivors in the low lactate group. The significant univariate indicators were included in the multivariate logistic regression analysis (Table 6). The mortality of patients with low lactate increased with age (OR = 0.966, 95% CI: 0.945–0.987, *P* = 0.002). The mortality of patients with malignancy was higher, and the difference was statistically significant (OR = 0.148, 95% CI: 0.061–0.359, *P* < 0.001). Required vasoactive drugs (OR = 4.745, 95% CI: 1.876–11.999, *P* = 0.001), glucocorticoid (OR = 2.579, 95% CI: 1.321–5.034, *P* = 0.005), and mechanical ventilation (OR = 2.926, 95% CI: 1.151–7.442, *P* = 0.024) and CRRT (OR = 3.079, 95% CI: 1.435–6.607, *P* = 0.004) had significant effects on the mortality of patients with low lactate in the early stage. In addition, LDH also had an impact on the mortality of patients in the low lactate group, with a statistical difference (OR = 0.999, 95% CI: 0.999–0.9998, *P* = 0.006). Statistical significance was not observed about fluid resuscitation volume in any of the time points mentioned above. But compared with the patients who met the standard fluid resuscitation volume in the first 6 hours, the mortality was higher in patients who failed to meet the standard, and the difference was statistically significant (OR = 22.621, 95% CI: 2.987–171.333, *P* = 0.003). For all participants, time-weighted lactate level was positively correlated with the amount of fluid resuscitation at each time point within 3H, 6H, 12H, and 24H after ICU admission (Figure 2), that is, the amount of fluid resuscitation increased with the increase of lactate level within 24 hours after ICU admission.

## 4. Discussion

This study confirmed the association between lactate level and mortality in patients with sepsis and septic shock again. The cutoff value of LacTW was 1.975 mmol/L, which is very close to the critical value of lactate in sepsis 3.0 [14]. However, the initial lactate value in ICU had no significant effect on mortality. It has been proved that time-weighted lactate is superior to static indices of lactate concentration and has significant independent predictive value of outcome in critically ill patients [19–21]. Our result reinforces the notion that, as a dynamic parameter, time-weighted lactate is a better proxy for early lactate levels than the initial lactate value in sepsis.

The definition of septic shock includes two indispensable components: elevated lactate levels and the need for vasoactive agents to maintain mean arterial pressure ≥65 mmHg after adequate fluid resuscitation. So, we may miss patients in the early stages of sepsis. In a multicenter study, patients with only refractory hypotension with normal lactate levels had a prevalence of 21.9% and a mortality of 29.6%. The morbidity and mortality of patients with hyperlactatemia and normal blood pressure were 31.6% and 27.5%, respectively [22, 23]. In this study, the mortality rate of patients with early sepsis and septic shock whose LacTW was less than 1.975 mmol/L for the first 24 hours after admission to ICU was 38.1%. Therefore, the severity of the disease cannot be misjudged by the absence of lactate level increasing, such inappropriate neglect may affect the timeliness and adequacy of specific treatment interventions, which may ultimately affect patient outcomes. That is, lactate is not omnipotent.

Therefore, the causality of lactate levels and mortality remains to be further verified. This is a clinical observational study, and the causality is less convincing than RCTs. Because doctors do not make decisions randomly but based on changes in the patient's condition. So, in this case, the relationship between lactate level and mortality is not a simple causal relationship. High lactate level may cause clinicians to attach great importance to it, and lactate level may decrease after treatment. In contrast, normal lactate level may lead to clinicians' neglect, thus missing the opportunity for treatment. In fact, the inference of causality of the longitudinal data can be further verified according to the method of Zhang et al. [24].

Why is lactate not elevated or delayed in some patients with septic shock despite circulatory failure in the early stage? We found that lactate levels increased in patients with liver, kidney, and coagulation dysfunction (especially in patients with renal insufficiency), hypotension, and high APACHE II score. Lactate remains the product of its production and elimination. Tissue hypoxia can lead to impaired mitochondrial function or microcirculation dysfunction, and lactate is overproduced and underutilized [25, 26]. Lactate can also be produced under aerobic conditions. It can be used as an energy source, messenger molecules, and gluconeogenesis precursors by various cells, tissues, organs, and the whole body to regulate body metabolism. Lactic acid is not a mark of hypoxia [8, 27, 28]. And lactate is mainly cleared in the liver and kidney, among which the liver is responsible for 70% of the systemic clearance rate. Studies have shown that patients with acute liver failure will lead to accelerated liver glycolysis and reduced gluconeogenesis, resulting in increased lactic acid levels [29, 30]. But in our study, renal insufficiency and creatinine level were positively correlated with lactic acid level. On the one hand, renal insufficiency leads to decreased lactate clearance rate; on the other hand, acute kidney injury is also a severe complication of sepsis, and recent studies have shown that lactic acid can induce immunosuppression by inducing lymphocyte apoptosis in septic acute kidney injury [31]. According to the results of this study, patients in the low-lactate group had a lower rate of organ dysfunction, so we can speculate that due to the lower incidence of early organ dysfunction, lactate levels are not increased or delayed in the early stage.

To avoid inappropriate neglect of such patients with sepsis who may have clinical deterioration but do not have elevated lactate levels in the early stage, we further analyzed that age, malignancy, and requirement of special treatments such as mechanical ventilation, CRRT, vasoactive drugs, and glucocorticoid had statistically different effects on mortality in the low lactate group. The fact that these indicators reflected disease severity has an impact on mortality is no surprise, which had been well established. But interestingly, we found that whether fluid resuscitation volume reached the standard of 30 ml/kg at 6H was an independent risk factor for predicting mortality in the low lactate group. This finding may suggest that low lactate levels cause clinicians to neglect, leading to inadequate or delayed treatment, and even further clinical deterioration of patients.

The surviving sepsis campaign guidelines recommend that in the resuscitation from sepsis-induced hypoperfusion (hypotension or lactic acidosis), at least 30 mL/kg of IV crystalloid fluid should be given within the first 3 hours [16, 32, 33]. A retrospective study has shown that sepsis patients who do not receive the recommended 30 ml/kg intravenous fluids within 3 hours have an increased risk for in-hospital mortality, delayed hypotension, and increased ICU length of stay. Moreover, elderly, the obese, male, patients with a history of heart failure, and end-stage renal disease are unlikely to achieve the standard in the first 3 hours [34]. However, few studies paid attention to fluid resuscitation in sepsis patients without increased lactate levels or the effect of lactate levels on fluid resuscitation.

In our study, there was no significant difference in baseline data including age, gender, weight, and underlying diseases between the low lactate group and the high lactate group. However, there were statistical differences in the fluid resuscitation volume at the time point of 3H, 6H, 12H, and 24H within the first 24 hours after ICU admission, and LacTW is positively correlated with fluid resuscitation volume. The low-lactate group had fewer patients who achieved the required fluid resuscitation and had a higher mortality rate than the high-lactate group. Therefore, the adequacy of fluid resuscitation should not be ignored because of the low lactic acid level. But more fluid resuscitation volume did not translate into lower mortality. Rivers and colleagues reported early goal-directed therapy (EGDT) to guide fluid resuscitation through continuous monitoring of prespecified physiological goals in 2001 [35]. However, several multicenter randomized controlled trials subsequently showed that EGDT has no significant advantage in improving mortality and organ dysfunction compared with ordinary care [23]. In addition, studies have shown that 67% of patients undergoing resuscitation with EGDT protocol had clinical evidence of fluid overload after 24 hours, and 48% of them had the characteristics of continuous fluid overload on the third day of hospitalization [36]. And positive fluid balance is independently associated with an increased risk of organ dysfunction and death in critically ill patients [37, 38]. So, in the early stage of sepsis, adequate fluid resuscitation should be performed as soon as possible, while restricted fluid resuscitation should be advocated in the later stage, rather than the more the better. Although guidelines currently recommend an initial fluid resuscitation volume of 30 ml/kg, the specific amount of fluid resuscitation required should vary from person to person. Dynamic monitoring of vital signs, microcirculation, fluid responsiveness, and other indicators is needed to adjust the patient's fluid balance dynamically.

As for the timing of early fluid resuscitation, the latest sepsis bundle recommends starting fluid resuscitation within 1 hour, and the volume is required to reach the standard within 3 hours [33, 39]. Differently, in this study, the time point of getting the standard of fluid resuscitation volume affecting mortality was 6 hours, it may be explained by the inappropriate neglect of clinicians caused by low lactate level, which not only affected the adequacy of fluid resuscitation volume but also affected the timeliness.

### 4.1. Limitations and Future Research

This study has several limitations. First, it is a single-center retrospective observational analysis, and all the clinical data were collected in one hospital. Second, since we focus on patients with sepsis or septic shock after entering ICU, the clinical index and treatment in the emergency department were not obtained. Third, patients with ICU hospitalization of fewer than 24 hours were excluded. Most of these patients died quickly after entering ICU, or their families gave up further treatment, which may lead to selection bias. In addition, this study did not specifically analyze the impact of the infection sources, pathogen, the type and dose of vasoactive drugs, and fluid type of resuscitation on mortality, which can be further followed up in subsequent studies.

## 5. Conclusions

The lactate level in the early stage of sepsis is related to organ function and prognosis. Compared with the initial lactate value, LacTW showed a more significant prognostic effect in patients with sepsis. Mortality in patients with no elevated lactate level in the early stage was associated with advanced age, malignancy, and the need for supportive treatment, such as the use of vasoactive drugs, glucocorticoid, mechanical ventilation, and CRRT, and the mortality in this group was also associated with whether the target fluid resuscitation volume within 6 hours. So, it can be calculated that due to the lower incidence of early organ dysfunction, lactate levels are not increased or delayed in some septic shock patients despite circulatory failure in the early stage, thus affecting the alertness of medical workers and the timeliness and adequacy of fluid resuscitation, and finally affecting the prognosis of these patients with septic shock. It may serve as a warning to clinicians to avoid ignoring sepsis patients at potential risk for clinical deterioration and whose early lactate levels are not elevated.

## Figures and Tables

**Table 1:** The comparison of baseline data between hospital survivors with nonsurvivors.

Variables	Hospital nonsurvivors (*n* = 418)	Hospital survivors (*n* = 412)	*P* value
Male (%)	272 (65.1)	249 (60.4)	0.167^c^
Age (years)	66 (54–75)	58 (50–71)	<0.001^b^
Weight (kg)	64.5 (55–70)	65 (55–70)	0.169^b^
Hospital stay (days)	14 (6–26)	19 (13–31.75)	<0.001^b^
ICU stay (days)	8 (3–16)	8 (4–13)	0.903^b^
Drinking history, yes (%)	48 (11.5)	48 (11.7)	0.940^c^
Smoking history, yes (%)	57 (13.6)	49 (11.9)	0.452^c^
Underlying disease, yes (%)			
Hypertension	156 (37.3)	150 (36.4)	0.785^c^
Diabetes	90 (21.5)	89 (21.6)	0.980^c^
Chronic liver disease	22 (5.3)	25 (6.1)	0.616^c^
Chronic kidney disease	48 (11.5)	31 (7.5)	0.052^c^
Chronic respiratory disease	30 (7.2)	28 (6.8)	0.830^c^
Cardiovascular disease	93 (22.2)	68 (16.5)	0.036^c^
Autoimmune disease	37 (8.9)	27 (6.6)	0.215^c^
Malignancy	104 (24.9)	49 (11.9)	<0.001^c^
Cerebrovascular disease	76 (18.2)	60 (14.6)	0.222^c^
Types of underlying disease ≥ 2	190 (45.5)	151 (36.7)	0.010^c^
Use of vasoactive drugs (%)	400 (95.7)	321 (77.9)	<0.001^c^
Use of glucocorticoid (%)	190 (45.5)	98 (23.8)	<0.001^c^
Mechanical ventilation (%)	378 (90.4)	226 (54.9)	<0.001^c^
CRRT (%)	179 (42.8)	77 (18.7)	<0.001^c^
APACHE II score	23 (19–27)	17 (13–22)	<0.001^b^
SOFA score	10 (8–13)	8 (5.25–11)	<0.001^b^
GCS score	3 (3–13)	13.5 (3–15)	<0.001^b^

^a^ *t*-test and the corresponding data were expressed as mean ± SD. ^b^Nonparametric test: Mann-Whitney*U* test and the corresponding data were expressed as median (interquartile range). ^c^Chi-square tests and the corresponding data were expressed as numbers (percentages). CRRT, continuous renal replacement therapy; APACHE II, acute physiology and chronic health evaluation II; SOFA, sepsis-related organ failure assessment; GCS, Glasgow coma scale.

**Table 2:** Multivariate analysis of predictive factors for hospitalization mortality of the whole participants.

Variables	OR (95% CI)	*P* value
Age	1.022 (1.008–1.036)	0.002
Hospital stay	0.962 (0.950–0.973)	<0.001
APACHE II score	1.048 (1.007–1.092)	0.022
Serum phosphorus	1.292 (1.040–1.605)	0.021
PCT	0.990 (0.983–0.997)	0.007
LacTW	1.171 (1.012–1.355)	0.034
Malignancy	2.799 (1.677–4.673)	<0.001
Use of vasoactive drugs	0.261 (0.134–0.507)	<0.001
Use of glucocorticoid	0.391 (0.259–0.592)	<0.001
Mechanical ventilation	0.299 (0.170–0.526)	<0.001
CRRT	0.340 (0.212–0.546)	<0.001
Fluid resuscitation volume (6H) ≥30 mL/kg	0.192 (0.080–0.465)	<0.001

*Note.* The univariate significant indicators in Table 1 and supplementary Table 1 were included in the multivariate logistic regression analysis, but only the statistically significant indicators in the multivariate analysis are listed here. APACHE II, acute physiology and chronic health evaluation II; PCT, procalcitonin; LacTW, time-weighted lactate; CRRT, continuous renal replacement therapy; 6H means in the first 6 hours after ICU admission.

**Table 3:** Multivariate analysis of predictive factors for lactate level in sepsis patients.

	*B* value	95% CI of *B* value		*T*	*P* value
		Lower limit	Upper limit		
(Constant)	5.262	3.244	7.280	5.118	<0.001
APACHE II score	0.066	0.030	0.102	3.607	<0.001
SOFA score	0.026	−0.048	0.100	0.689	0.491
PLT	0.001	−0.001	0.003	0.697	0.486
PT	−0.001	−0.009	0.008	−0.174	0.862
APTT	0.016	0.006	0.026	3.058	0.002
TBIL	0.005	0.001	0.009	2.517	0.012
LDH	0.000	0.000	0.000	5.599	<0.001
eGFR	−0.005	−0.011	0.002	−1.493	0.136
CRE	−0.002	−0.003	0.000	−2.088	0.037
Refractory hypotension	−0.819	−1.238	−0.400	−3.837	<0.001
Use of vasoactive drugs	−0.273	−0.892	0.346	−0.865	0.387
Use of glucocorticoid	−0.391	−0.805	0.023	−1.855	0.064
Mechanical ventilation	0.095	−0.441	0.631	0.348	0.728
CRRT	−1.356	−1.826	−0.886	−5.660	<0.001
Chronic liver disease	0.736	−0.110	1.582	1.707	0.088
Chronic kidney disease	−0.923	−1.653	−0.194	−2.484	0.013
Autoimmune disease	0.311	−0.428	1.049	0.826	0.409
Malignancy	0.174	−0.319	0.668	0.694	0.488

APACHE II, acute physiology and chronic health evaluation II; SOFA, sepsis-related organ failure assessment; PLT, platelet count; PT, prothrombin time; APTT, activated partial thromboplastin time; TBIL, total bilirubin; LDH, lactate dehydrogenase; CRE, creatinine; eGFR, estimated glomerular filtration rate; CRRT, continuous renal replacement therapy. In the multiple linear regression model, adjusted *R*^2^ = 0.232, *F* = 14.940, *P* < 0.001.

**Table 4:** Baseline data and fluid resuscitation comparison of patients in the high lactate group and the low lactate group.

Characteristic	Low lactate (*n* = 394)	High lactate (*n* = 436)	*P* value
Baseline date			
Male (%)	244 (61.9)	277 (63.5)	0.633^b^
Age (years)	63 (52–73)	63 (51–73)	0.719^a^
Weight (kg)	65 (55–70)	65 (55–70)	0.875^a^
Hospital stay (days)	18 (11–30)	15 (8–27)	<0.001^a^
ICU stay (days)	8 (4–15)	7 (3–14)	0.018^a^
Drinking history, yes (%)	45 (11.4)	51 (11.7)	0.901^b^
Smoking history, yes (%)	51 (12.9)	55 (12.6)	0.887^b^
Underlying disease, yes (%)			
Hypertension	154 (39.1)	152 (34.9)	0.208^b^
Diabetes	84 (21.3)	95 (21.8)	0.870^b^
Chronic liver disease	16 (4.1)	31 (7.1)	0.058^b^
Chronic kidney disease	44 (11.2)	35 (8)	0.124^b^
Chronic respiratory disease	28 (7.1)	30 (6.9)	0.899^b^
Cardiovascular disease	78 (19.8)	83 (19)	0.782^b^
Autoimmune disease	27 (6.9)	37 (8.5)	0.378^b^
Malignancy	64 (16.2)	89 (20.4)	0.122^b^
Cerebrovascular disease	74 (18.8)	63 (14.4)	0.136^b^
Types of underlying disease ≥ 2	171 (43.4)	170 (39)	0.197^b^
Fluid resuscitation			
Fluid resuscitation volume (3H) (mL)	250 (150–525)	398 (200–720)	<0.001^a^
Fluid resuscitation volume (6H) (mL)	714.5 (495.8–1106)	967 (621.5–1480)	<0.001^a^
Fluid resuscitation volume (12H) (mL)	1627 (1160–2120)	1966 (1372.5–2673)	<0.001^a^
Fluid resuscitation volume (24H) (mL)	3249 (2530–4074)	3701 (2949–4937.5)	<0.001^a^
Fluid resuscitation volume (3H) ≥ 30 mL/kg (%)	4 (1.0)	7 (1.6)	0.458^b^
Fluid resuscitation volume (6H) ≥ 30 mL/kg (%)	39 (9.9)	64 (14.7)	0.037^b^
Fluid resuscitation volume (12H) ≥ 30 mL/kg (%)	141 (35.8)	231 (53.0)	<0.001^b^
Fluid resuscitation volume (24H) ≥ 30 mL/kg (%)	350 (88.8)	415 (95.2)	0.001^b^

^a^Nonparametric test: Mann-Whitney*U* test. And the corresponding data were expressed as median (interquartile range). ^b^Chi-square tests and the corresponding data were expressed as numbers (percentages). 3H, 6H, 12H, and 24H, mean the first 3 hours, 6 hours, 12 hours, and 24 hours after ICU admission.

**Table 5:** Organ dysfunction and clinical outcome comparison of patients in the high lactate group and the low lactate group.

	Low lactate *n* = 394	High lactate *n* = 436	*P* value
Hospital stay (days)	18 (11–30)	15 (8–27)	<0.001^a^
ICU stay (days)	8 (4–15)	7 (3–14)	0.018^a^
Hospital mortality (%)	150 (38.1)	268 (61.5)	<0.001^b^
APACHE II score	18 (13–23)	22 (18–26)	<0.001^a^
SOFA score	8 (5–11)	10 (8–13)	<0.001^a^
PT (S)	15.6 (14.4–17.3)	17.4 (15.6–20.4)	<0.001^a^
APTT (S)	42.2 (37.0–49.5)	45.3 (38.8–54.4)	<0.001^a^
TBIL (*μ*mol/L)	16.95 (10.80–30.64)	23 (13.25–40.97)	<0.001^a^
CRE (*μ*mol/L)	100.2 (61.6–205.5)	126.6 (79.15–244.75)	0.001^a^
Refractory hypotension	139 (35.3)	233 (53.4)	<0.001^b^
CRRT	90 (22.8)	166 (38.1)	<0.001^b^

^a^Nonparametric test: Mann-Whitney*U* test. And the corresponding data were expressed as median (interquartile range). ^b^Chi-square tests and the corresponding data were expressed as numbers (percentages). APACHE II, acute physiology and chronic health evaluation II; SOFA, sepsis-related organ failure assessment; PT, prothrombin time; APTT, activated partial thromboplastin time; TBIL, total bilirubin; CRE, creatinine; CRRT, continuous renal replacement therapy.

**Table 6:** Multivariate analysis of predictive factors for hospital mortality of patients in the low lactate group.

Variables	OR (95% CI)	*P* value
Age	0.966 (0.945–0.987)	0.002
ICU stay	1.006 (0.985–1.028)	0.575
Chronic kidney disease	0.498 (0.174–1.427)	0.194
Cardiovascular disease	0.930 (0.406–2.127)	0.863
Malignancy	0.148 (0.061–0.359)	<0.001
Cerebrovascular disease	0.600 (0.256–1.407)	0.240
Types of underlying disease ≥ 2	0.932 (0.417–2.086)	0.864
Use of vasoactive drugs	4.745 (1.876–11.999)	0.001
Use of glucocorticoid	2.579 (1.321–5.034)	0.005
Mechanical ventilation	2.926 (1.151–7.442)	0.024
CRRT	3.079 (1.435–6.607)	0.004
APACHE II score	0.964 (0.904–1.027)	0.257
SOFA score	1.084 (0.963–1.220)	0.183
GCS score	1.065 (0.978–1.159)	0.148
Hb	1.006 (0.993–1.018)	0.385
RDW	0.956 (0.888–1.030)	0.235
TBIL	0.996 (0.988–1.005)	0.436
ALT	1.001 (1.000–1.002)	0.060
LDH	0.999 (0.999–0.9998)	0.006
Serum potassium	0.885 (0.601–1.303)	0.537
Serum sodium	0.982 (0.943–1.022)	0.364
HCO3	0.996 (0.944–1.051)	0.893
PCT	1.003 (0.995–1.011)	0.454
Fluid resuscitation volume (3H)	1.000 (0.999–1.002)	0.748
Fluid resuscitation volume (6H)	0.999 (0.997–1.000)	0.151
Fluid resuscitation volume (12H)	1.000 (0.999–1.001)	0.648
Fluid resuscitation volume (24H)	1.000 (1.000–1.001)	0.067
Albumin infusion volume (12H)	0.977 (0.946–1.009)	0.157
Albumin infusion volume (24H)	1.021 (0.998–1.045)	0.078
Fluid resuscitation volume (6H) ≥30 mL/kg	22.621 (2.987–171.333)	0.003
Fluid resuscitation volume (12H) ≥30 mL/kg	0.600 (0.247–1.457)	0.260

*Note.* The univariate significant indicators in Supplementary Table 2 were included in the multivariate logistic regression analysis. CRRT, continuous renal replacement therapy; APACHE II, acute physiology and chronic health evaluation II; SOFA, sepsis-related organ failure assessment; GCS, Glasgow coma scale; Hb, hemoglobin; RDW, red blood cell distribution width; TBIL, total bilirubin; ALT, alanine aminotransferase; LDH, lactate dehydrogenase; HCO3, serum bicarbonate concentration; PCT, procalcitonin.

## Data Availability

The data used to support the findings of this study are available from the corresponding author upon request.

## Supplementary Materials

Supplementary Materials The comparisons of clinical characteristics between the nonsurvivors group and the survivors group in all participants and the low lactate group were shown in Supplementary Table 1 and Supplementary Table 2.

## References

[1] Fleischmann-Struzek C., Mellhammar L., Rose N. (2020). Incidence and mortality of hospital- and ICU-treated sepsis: results from an updated and expanded systematic review and meta-analysis. *Intensive Care Medicine*.

[2] Rhee C., Dantes R., Epstein L. (2017). Incidence and trends of sepsis in US hospitals using clinical vs. claims data, 2009-2014. *JAMA*.

[3] Iwashyna T. J., Cooke C. R., Wunsch H., Kahn J. M. (2012). Population burden of long-term survivorship after severe sepsis in older Americans. *Journal of the American Geriatrics Society*.

[4] Fleischmann C., Scherag A., Adhikari N. K. J. (2016). Assessment of global incidence and mortality of hospital-treated sepsis. Current estimates and limitations. *American Journal of Respiratory and Critical Care Medicine*.

[5] Liu G. L., Lv H. J., An Y. L., Wei X. X., Yi X. M., Yi H. M. (2017). Early lactate levels for prediction of mortality in patients with sepsis or septic shock: a meta-analysis. *International Journal of Clinical and Experimental Medicine*.

[6] Casserly B., Phillips G. S., Schorr C. (2015). Lactate measurements in sepsis-induced tissue hypoperfusion: results from the Surviving Sepsis Campaign database. *Critical Care Medicine*.

[7] Borthwick H. A., Brunt L. K., Mitchem K. L., Chaloner C. (2012). Does lactate measurement performed on admission predict clinical outcome on the intensive care unit? A concise systematic review. *Annals of Clinical Biochemistry*.

[8] Levy B. (2006). Lactate and shock state: the metabolic view. *Current Opinion in Critical Care*.

[9] Bakker J., Postelnicu R., Mukherjee V. (2020). Lactate: where are we now?. *Critical Care Clinics*.

[10] Suetrong B., Walley K. R. (2016). Lactic acidosis in sepsis: it’s not all anaerobic. *Chest*.

[11] Hernandez G., Bellomo R., Bakker J. (2019). The ten pitfalls of lactate clearance in sepsis. *Intensive Care Medicine*.

[12] Vincent J. L., Bakker J. (2021). Blood lactate levels in sepsis: in 8 questions. *Current Opinion in Critical Care*.

[13] Jansen T. C., van Bommel J., Schoonderbeek F. J. (2010). Early lactate-guided therapy in intensive care unit patients: a multicenter, open-label, randomized controlled trial. *American Journal of Respiratory and Critical Care Medicine*.

[14] Singer M., Deutschman C. S., Seymour C. W. (2016). The third international consensus definitions for sepsis and septic shock (Sepsis-3). *JAMA*.

[15] Sterling S. A., Puskarich M. A., Glass A. F., Guirgis F., Jones A. E. (2017). The impact of the sepsis-3 septic shock definition on previously defined septic shock patients. *Critical Care Medicine*.

[16] Rhodes A., Evans L. E., Alhazzani W. (2016). Surviving sepsis campaign: international guidelines for management of sepsis and septic shock. *Intensive Care Medicine*.

[17] Nichol A., Bailey M., Egi M. (2011). Dynamic lactate indices as predictors of outcome in critically ill patients. *Critical Care*.

[18] Finney S. J., Zekveld C., Elia A., Evans T. W. (2003). Glucose control and mortality in critically ill patients. *JAMA*.

[19] Nguyen H. B. (2011). Lactate in the critically ill patients: an outcome marker with the times. *Critical Care*.

[20] Bosso G., Mercurio V., Diab N. (2021). Time-weighted lactate as a predictor of adverse outcome in acute heart failure. *ESC Heart Failure*.

[21] Alam A., Gupta S. (2021). Lactate measurements and their association with mortality in pediatric severe sepsis in India: evidence that 6-hour level performs best. *Journal of Intensive Care Medicine*.

[22] Peake S. L., Delaney A., Bailey M. (2014). Goal-directed resuscitation for patients with early septic shock. *New England Journal of Medicine*.

[23] Osborn T. M. (2017). Severe sepsis and septic shock trials (ProCESS, ARISE, ProMISe): what is optimal resuscitation?. *Critical Care Clinics*.

[24] Zhang Z., Jin P., Feng M. (2022). Causal inference with marginal structural modeling for longitudinal data in laparoscopic surgery: a technical note. *Laparoscopic, Endoscopic and Robotic Surgery*.

[25] Kraut J. A., Madias N. E. (2014). Lactic acidosis. *New England Journal of Medicine*.

[26] Ince C. (2005). The microcirculation is the motor of sepsis. *Critical Care*.

[27] Brooks G. A. (2018). The science and translation of lactate shuttle theory. *Cell Metabolism*.

[28] Brooks G. A. (2020). Lactate as a fulcrum of metabolism. *Redox Biology*.

[29] Jeppesen J. B., Mortensen C., Bendtsen F., Moller S. (2013). Lactate metabolism in chronic liver disease. *Scandinavian Journal of Clinical and Laboratory Investigation*.

[30] Clemmesen J. O., Høy C. E., Kondrup J., Ott P. (2000). Splanchnic metabolism of fuel substrates in acute liver failure. *Journal of Hepatology*.

[31] Xu J., Ma X., Yu K. (2021). Lactate up-regulates the expression of PD-L1 in kidney and causes immunosuppression in septic Acute Renal Injury. *Journal of Microbiology, Immunology, and Infection*.

[32] Dellinger R. P., Levy M. M., Rhodes A. (2012). Sepsis Campaign Guidelines Committee including the Pediatric, “Surviving sepsis campaign: international guidelines for management of severe sepsis and septic shock. *Critical Care Medicine*.

[33] Evans L., Rhodes A., Alhazzani W. (2021). Surviving sepsis campaign: international guidelines for management of sepsis and septic shock 2021. *Intensive Care Medicine*.

[34] Kuttab H. I., Lykins J. D., Hughes M. D. (2019). Evaluation and predictors of fluid resuscitation in patients with severe sepsis and septic shock. *Critical Care Medicine*.

[35] Rivers E., Nguyen B., Havstad S. (2001). Early goal-directed therapy in the treatment of severe sepsis and septic shock. *New England Journal of Medicine*.

[36] Kelm D. J., Perrin J. T., Cartin-Ceba R., Gajic O., Schenck L., Kennedy C. C. (2015). Fluid overload in patients with severe sepsis and septic shock treated with early goal-directed therapy is associated with increased acute need for fluid-related medical interventions and hospital death. *Shock*.

[37] Claure-Del Granado R., Mehta R. L. (2016). Fluid overload in the ICU: evaluation and management. *BMC Nephrology*.

[38] Bouchard J., Soroko S. B., Chertow G. M. (2009). Fluid accumulation, survival and recovery of kidney function in critically ill patients with acute kidney injury. *Kidney International*.

[39] Levy M. M., Evans L. E., Rhodes A. (2018). The surviving sepsis campaign bundle: 2018 update. *Intensive Care Medicine*.

