# Supplementary material for: Risk Factors for Mortality in Sepsis Patients without Lactate Levels Increasing Early
Source: Emerg Med Int. 2023 Feb 24;2023:6620157. doi: 10.1155/2023/6620157 (PMC9984259; doi:10.1155/2023/6620157)
Supplement: Supplementary Materials — The comparisons of clinical characteristics between the nonsurvivors group and the survivors group in all participants and the low lactate group were shown in Supplementary Table 1 and Supplementary Table 2. [file 6620157.f1.zip › Supplymentary Table 1.doc]

**Supplementary Table 1. Clinical characteristics of hospital survivors and non-survivors**

| **Variables** | **Hospital non-survivors(n=418)** | **Hospital survivors(n=412)** | ***P* value** |
| --- | --- | --- | --- |
| **MAP (0H), mmHg** | 81.83(67.3-96.42) | 81(67-95.25) | 0.941 ^b^ |
| **WBC, ×10^9^/L** | 12.31(7.33-17.62) | 12.16(7.47-18.3) | 0.600 ^b^ |
| **Neutrophil ratio, %** | 89.12(81.88-93.43) | 89.72(83.62-93.67) | 0.182 ^b^ |
| **Hb, g/L** | 99.81±26.84 | 107.43±27.99 | <0.001^a^ |
| **PLT, ×10^9^/L** | 125.5(63-197.25) | 129.5(72-196) | 0.598 ^b^ |
| **RDW, fl** | 48.3(44.68-53.15) | 45.35(42.4-45.35) | <0.001 ^b^ |
| **HCT, %** | 30.11±8.17 | 31.93±8.2 | 0.001 ^a^ |
| **PT, S** | 16.7(15.1-19.4) | 16.2(14.7-18.1) | 0.002 ^b^ |
| **APTT, S** | 43.85(37.68-53.30) | 43.60(37.70-51.10) | 0.520 ^b^ |
| **INR** | 1.35(1.19-1.63) | 1.29(1.15-1.50) | 0.001 ^b^ |
| **PTA, %** | 61.61±20.52 | 65.91±19.48 | 0.002 ^a^ |
| **TBIL, μmol/L** | 18.88(11.4-33.00) | 21.21(12.30-36.43) | 0.131 ^b^ |
| **ALT, U/L** | 36(19-82) | 35.5(21-90.5) | 0.996 ^b^ |
| **AST, U/L** | 59(30-137) | 46(29.5-121.5) | 0.072 ^b^ |
| **CRE, μmol/L** | 124(69.7-256.4) | 113.1(7.98-21.1) | 0.169 ^b^ |
| **BUN, mmol/L** | 13.14(7.98-21.1) | 10.2(6.8-16.15) | <0.001 ^b^ |
| **LDH, U/L** | 634.5(341-1310.93) | 461(194.5-846.5) | <0.001 ^b^ |
| **CK, U/L** | 198(59-933) | 166.5(60-679) | 0.259 ^b^ |
| **CK-MB, U/L** | 19(8-41) | 16(7-31) | 0.026 ^b^ |
| **eGFR, ml/(min·1.73m^2^)** | 51(21-95) | 58.5(28-100) | 0.032 ^b^ |
| **Serum potassium, mmol/L** | 4.14(3.68-4.77) | 3.86(3.49-4.40) | <0.001 ^b^ |
| **Serum sodium, mmol/L** | 129.05(135.5-143.8) | 138(134.25-141.3) | 0.214 ^b^ |
| **Serum calcium, mmol/L** | 2.02(1.87-2.19) | 2.03(1.89-2.18) | 0.986 ^b^ |
| **Serum phosphorus, mmol/L** | 1.13(0.77-1.73) | 0.96(0.65-1.36) | <0.001 ^b^ |
| **HCO3, mmol/L** | 20.43±6.31 | 20.2±5.91 | 0.584 ^a^ |
| **Troponin, ng/mL** | 0.105(0.02-0.52) | 0.54(0.01-0.24) | <0.001 ^b^ |
| **Myoglobin, ng/mL** | 351.2(107-691) | 223(71-420.8) | <0.001 ^b^ |
| **PCT, ng/mL** | 7.08(1.18-30.95) | 13.89(2.28-58.08) | <0.001 ^b^ |
| **CRP, mg/L** | 113.71(43.7-180.69) | 131.45(49.25-184.83) | 0.320 ^b^ |
| **Initial** **blood lactate, mmol/L** | 2.755(1.7-5.3) | 2.1(1.325-3.495) | <0.001 ^b^ |
| **LacTW, mmol/L** | 2.345(1.61-4.21) | 1.765(1.245-2.665) | <0.001 ^b^ |
| **Fluid resuscitation volume (3H), mL** | 300(154.5-600) | 340(18.5-650) | 0.139 ^b^ |
| **Fluid resuscitation volume (6H), mL** | 800(510-1192.5) | 887(570-1440) | 0.005 ^b^ |
| **Fluid resuscitation volume (12H), mL** | 1715(1263.75-2352.5) | 1841(1305.5-2478) | 0.084 ^b^ |
| **Fluid resuscitation volume (24H), mL** | 3376(2679.5-4413.0) | 3583(2750-4676) | 0.103 ^b^ |
| **Fluid resuscitation volume (3H) ≥30mL/kg** | 6(1.4) | 5(1.2) | 0.780 ^c^ |
| **Fluid resuscitation volume (6H) ≥30mL/kg** | 32(7.7) | 71(17.2) | <0.001^c^ |
| **Fluid resuscitation volume (12H) ≥30mL/kg** | 179(42.8) | 193(46.8) | 0.244 ^c^ |
| **Fluid resuscitation volume (24H) ≥30mL/kg** | 388(92.8) | 377(91.5) | 0.480 ^c^ |
| **Albumin infusion volume (3H), g** | 0(0-10) | 0(0-13.75) | 0.261 ^b^ |
| **Albumin infusion volume (6H), g** | 10(0-20) | 10(0-20) | 0.371 ^b^ |
| **Albumin infusion volume (12H), g** | 20(10-30) | 20(10-30) | 0.481 ^b^ |
| **Albumin infusion volume (24H), g** | 40(20-60) | 40(20-60) | 0.889 ^b^ |

a t-test and the corresponding data were expressed as mean ± SD.

b non-parametric test: Mann-Whitney U-test and the corresponding data were expressed as median (interquartile range).

c chi-square tests and the corresponding data were expressed as numbers (percentages).

MAP, mean arterial pressure; WBC, white blood cell count; Hb, hemoglobin; PLT, platelet count; RDW, red blood cell distribution width; HCT, hematocrit; PT, prothrombin time; APTT, activated partial thromboplastin time; INR, international normalized ratio; PTA, prothrombin time activity; TBIL, total bilirubin; ALT, alanine aminotransferase; AST, aspartate aminotransferase; LDH, lactate dehydrogenase; BUN, urea nitrogen; CRE, creatinine; eGFR, estimated glomerular filtration rate; CK, creatine kinase; CK-MB, creatine kinase isoenzyme; HCO3, serum bicarbonate concentration; PCT, procalcitonin; CRP, C-reactive protein; LacTW, time-weighted lactate.

0H means the time point when entering ICU. 3H,6H,12H, and 24H, mean the first 3 hours,6 hours,12 hours, and 24 hours after ICU admission.
