# Supplementary material for: Risk Factors for Mortality in Sepsis Patients without Lactate Levels Increasing Early
Source: Emerg Med Int. 2023 Feb 24;2023:6620157. doi: 10.1155/2023/6620157 (PMC9984259; doi:10.1155/2023/6620157)
Supplement: Supplementary Materials — The comparisons of clinical characteristics between the nonsurvivors group and the survivors group in all participants and the low lactate group were shown in Supplementary Table 1 and Supplementary Table 2. [file 6620157.f1.zip › Supplymentary Table 2.doc]

**Supplementary Table 2. Clinical characteristics of hospital survivors and non-survivors in the low lactate group**

| **Characteristics** | **Hospital non-survivors(n=150)** | **Hospital survivors**  **(n=244)** | ***P* value** |
| --- | --- | --- | --- |
| **Male(%)** | 97(64.7) | 147(60.2) | 0.380 ^c^ |
| **Age, years** | 68(56-78) | 58(49-71) | <0.001 ^b^ |
| **Weight, kg** | 65(55-70) | 65(55-70) | 0.497 ^b^ |
| **Hospital stay, days** | 18(10-28) | 18.5(12-30) | 0.114 ^b^ |
| **ICU stay, days** | 11(6-18) | 7(4-13) | <0.001 ^b^ |
| **Drinking history, yes (%)** | 17(11.3) | 28(11.5) | 0.966 ^c^ |
| **Smoking history, yes (%)** | 20(13.3) | 31(12.7) | 0.857 ^c^ |
| **underlying disease, yes (%)** |  |  |  |
| **Hypertension** | 64(42.7) | 90(36.9) | 0.253 ^c^ |
| **Diabetes** | 31(20.7) | 53(21.7) | 0.804 ^c^ |
| **Chronic liver disease** | 3(2) | 13(5.3) | 0.104 ^c^ |
| **Chronic kidney disease** | 24(16) | 20(8.2) | 0.017 ^c^ |
| **Chronic respiratory disease** | 12(8) | 16(6.6) | 0.588 ^c^ |
| **Cardiovascular disease** | 39(26) | 39(16) | 0.015 ^c^ |
| **Autoimmune disease** | 10(6.7) | 17(7) | 0.909 ^c^ |
| **Malignancy** | 44(29.3) | 20(8.2) | <0.001 ^c^ |
| **Cerebrovascular disease** | 37(24.7) | 37(15.2) | 0.019 ^c^ |
| **types of underlying disease ≥2** | 82(54.7) | 89(36.5) | <0.001 ^c^ |
| **Use of vasoactive drugs (%)** | 140(93.3) | 173(70.9) | <0.001 ^c^ |
| **Use of glucocorticoid (%)** | 64(42.7) | 48(19.7) | <0.001 ^c^ |
| **Mechanical ventilation (%)** | 132(88) | 122(50) | <0.001 ^c^ |
| **CRRT (%)** | 52(34.7) | 38(15.6) | <0.001 ^c^ |
| **APACHE Ⅱ score** | 21.45±6.8 | 16.41±6.72 | <0.001^a^ |
| **SOFA score** | 9(6-12) | 7(5-10) | <0.001 ^b^ |
| **GCS score** | 4.5(3-13) | 14(3.5-15) | <0.001 ^b^ |
| **MAP (0H), mmHg** | 88.75±19.56 | 85.60±20.37 | 0.131 ^a^ |
| **Fluid resuscitation volume (3H), mL** | 225(127-400) | 284(172.5-600) | 0.002 ^b^ |
| **Fluid resuscitation volume (6H), mL** | 650(472-918) | 748.5(508.5-1300) | <0.001 ^b^ |
| **Fluid resuscitation volume (12H), mL** | 1422.5(1070-1910) | 1750(1216.5-2290) | <0.001 ^b^ |
| **Fluid resuscitation volume (24H), mL** | 2997.5(2351-3561) | 3493(2634-4341.5) | <0.001 ^b^ |
| **Fluid resuscitation volume (3H) ≥30mL/kg** | 0(0) | 4(1.6) | 0.115 ^c^ |
| **Fluid resuscitation volume (6H) ≥30mL/kg** | 2(1.3) | 37(15.2) | <0.001 |
| **Fluid resuscitation volume (12H) ≥30mL/kg** | 41(27.3) | 100(41) | 0.006 ^c^ |
| **Fluid resuscitation volume (24H) ≥30mL/kg** | 134(89.3) | 216(88.5) | 0.805 ^c^ |
| **Albumin infusion volume** **(3H), g** | 0(0-10) | 0(0-10) | 0.153 ^b^ |
| **Albumin infusion volume (6H), g** | 10(0-20) | 10(0-20) | 0.001 ^b^ |
| **Albumin infusion volume (12H), g** | 20(0-30) | 20(10-30) | 0.060 ^b^ |
| **Albumin infusion volume (24H), g** | 30(20-50) | 40(20-60) | 0.014 ^b^ |
| **WBC, ×10^9^/L** | 12.735(8.14-16.34) | 12.1(8.925-17.45) | 0.904 ^b^ |
| **Neutrophil ratio, %** | 90.65(85.4-93.61) | 90.6(85.075-93.8) | 0.888 ^b^ |
| **Hb, g/L** | 96(80-117) | 101(85-122) | 0.050 ^b^ |
| **PLT, ×10^9^/L** | 141.5(90-228) | 138.5(73-205) | 0.353 ^b^ |
| **RDW, fl** | 49.15(43.9-54.2) | 45.5(42.05-49.75) | <0.001 ^b^ |
| **HCT, %** | 28.45(24.5-35.6) | 30.25(25.6-36.2) | 0.202 ^b^ |
| **PT, S** | 15.7(14.5-17.5) | 15.6(14.4-17.2) | 0.671 ^b^ |
| **APTT, S** | 40.85(36.7-49.3) | 42.7(37.2-49.65) | 0.298 ^b^ |
| **INR** | 1.24(1.12-1.43) | 1.25(1.115-1.39) | 0.580 ^b^ |
| **PTA, %** | 69.92±17.21 | 70.30±17.88 | 0.833 ^a^ |
| **TBIL, μmol/L** | 13.84(9.4-22.8) | 18.9(12.05-35.05) | <0.001 ^b^ |
| **ALT, U/L** | 27.5(17-51) | 34(20-69) | 0.022 ^b^ |
| **AST, U/L** | 39.5(27-75) | 42(26-92) | 0.385 ^b^ |
| **CRE, μmol/L** | 88.7(61.2-256) | 107.55(63.3-198.7) | 0.703 ^b^ |
| **BUN, mmol/L** | 12.12(6.6-19.1) | 9.96(6.46-16.195) | 0.096 ^b^ |
| **LDH, U/L** | 626.5(327-1033) | 415(275.5-711.5) | 0.001 ^b^ |
| **CK, U/L** | 127(43-779) | 153(50.5-622) | 0.773 ^b^ |
| **CKMB, U/L** | 14(4-28) | 13(6-27) | 0.848 ^b^ |
| **eGFR, ml/(min·1.73m^2^)** | 69(20-99) | 61.5(30.5-107.5) | 0.417 ^b^ |
| **Serum potassium, mmol/L** | 4.045(3.64-4.75) | 3.87(3.465-4.425) | 0.012 ^b^ |
| **Serum sodium, mmol/L** | 138.45(134.8-143.1) | 137.35(134-140.7) | 0.037 ^b^ |
| **Serum calcium, mmol/L** | 1.99(1.86-2.16) | 2.03(1.89-2.17) | 0.225 ^b^ |
| **Serum phosphorus, mmol/L** | 0.955(0.65-1.45) | 0.96(0.69-1.335) | 0.855 ^b^ |
| **HCO3, mmol/L** | 22.55(19.1-25.6) | 20.55(17.25-24.35) | 0.005 ^b^ |
| **Troponin, ng/mL** | 0.058(0.01-0.2) | 0.03(0.01-0.173) | 0.078 ^b^ |
| **Myoglobin, ng/mL** | 232(68-411) | 160.5(53.5-386.3) | 0.144 ^b^ |
| **PCT, ng/mL** | 2.59(0.49-11.17) | 8.485(1.495-34.245) | <0.001 ^b^ |
| **CRP, mg/L** | 120.08(59.34-176.57) | 133.69(56.94-188.89) | 0.333 ^b^ |
| **Initial blood lactate, mmol/L** | 1.515(1.12-1.98) | 1.5(1.1-2.1) | 0.744 ^b^ |

^a^ t-test. and the corresponding data were expressed as mean ± SD.

^b^ non-parametric test: Mann-Whitney U-test and the corresponding data were expressed as median (interquartile range).

^c^ chi-square tests and the corresponding data were expressed as numbers (percentages).

CRRT, continuous renal replacement therapy; APACHE Ⅱ, acute physiology and chronic health evaluation II; SOFA, sepsis-related organ failure assessment; GCS, Glasgow Coma Scale; MAP, mean arterial pressure; WBC, white blood cell count; Hb, hemoglobin; PLT, platelet count; RDW, red blood cell distribution width; HCT, hematocrit; PT, prothrombin time; APTT, activated partial thromboplastin time; INR, international normalized ratio; PTA, prothrombin time activity; TBIL, total bilirubin; ALT, alanine aminotransferase; AST, aspartate aminotransferase; LDH, lactate dehydrogenase; BUN, urea nitrogen; CRE, creatinine; eGFR, estimated glomerular filtration rate; CK, creatine kinase; CK-MB, creatine kinase isoenzyme; HCO3, serum bicarbonate concentration; PCT, procalcitonin; CRP, C-reactive protein.

0H means the time point when entering ICU. 3H,6H,12H, and 24H, mean the first 3 hours,6 hours,12 hours, and 24 hours after ICU admission.
